# Use of biomarkers in the context of orphan medicines designation in the European Union

**DOI:** 10.1186/1750-1172-9-13

**Published:** 2014-01-27

**Authors:** Stelios Tsigkos, Jordi Llinares, Segundo Mariz, Stiina Aarum, Laura Fregonese, Bozenna Dembowska-Baginska, Rembert Elbers, Pauline Evers, Tatiana Foltanova, Andre Lhoir, Ana Corrêa-Nunes, Daniel O’Connor, Albertha Voordouw, Kerstin Westermark, Bruno Sepodes

**Affiliations:** 1 Orphan Medicines Office, European Medicines Agency, 7 Westferry circus, E144HB London, UK; 2 Committee of Orphan Medicinal Products, European Medicines Agency, 7 Westferry circus, E144HB London, UK; 3 Faculty of Pharmacy, Comenius University, Bratislava, Slovak Republic; 4 Pharmacovigilance Department Bundesinstitut für Arzneimittel und Medizinprodukte Kurt-Georg-Kiesinger-Allee, 3 53175 Bonn, Germany; 5 Medicines Evaluation Board Forum Building Graadt van Roggenweg, 500 3531 AH Utrecht, Netherlands; 6 Department of Pharmacological Sciences - Faculty of Pharmacy, University of Lisbon, 1649-003 Lisboa, Portugal

**Keywords:** Orphan medicinal product designation, Distinct medical entity, Biomarker

## Abstract

The use of biomarkers within the procedures of the Committee of Orphan Medicinal Products (COMP) of the European Medicines Agency (EMA) is discussed herein. The applications for Orphan Medicinal Product designation in the EU are evaluated at two stages. At the time of orphan designation application, the file undergoes an assessment to establish whether the proposed condition is a distinct and serious condition affecting not more than 5 in 10,000 people in the EU, and whether the product is plausible as a therapy for that condition. In cases where therapies already exist, the significant benefit of the candidate product over existing therapies is also evaluated. The orphan criteria are reassessed at the time of marketing authorisation, so that marketing exclusivity for the product in the orphan medical condition can be granted. Within this context, biomarkers have been used in submissions in order to define an orphan condition and to justify that the criteria for orphan designation are met. The current work discusses specific examples from the experience of the COMP, where biomarkers have played a decisive role. Importantly, it identifies the proposal of sub-sets of non-rare conditions based on biomarkers as a challenging issue in the evaluation of applications. In particular two specific requirements for the candidate orphan medicines in relation to the biomarker-based subsets are highlighted: the “plausible link to the condition” and the “exclusion of effects outside the subset”.

## Background

The use of biomarkers, which are specific for medical conditions, is envisioned to accelerate the research and development and increase the success rate of pharmaceutical products [1]. This approach is characterised by increased specificity of therapeutic targets and delineation of specific populations for which a medicinal product has improved efficacy or improved safety. In the field of developing medicines in conditions that occur so rarely that pharmaceutical industry generally believe that the return on investment is not optimal (the so-called orphan medicinal products), this approach may consequently lead to an enrichment of the limited armamentarium to diagnose prevent or treat those conditions.

The Committee of Orphan Medicinal Product (COMP) of the European Medicines Agency (EMA) has always faced challenges when evaluating submissions for orphan designation. The COMP has been assessing the use of biomarkers by sponsors of candidate orphan products in three main areas:

•to define the distinct medical condition or a valid sub-set for the designation (orphan condition as described in guideline ENTR6283/Rev03) [2]

•to justify the intention to diagnose, prevent or treat a condition with a product, (as per the provisions of Article 3(1)(a) of Regulation (EC) No 141/2000 [3])

•to determine significant benefit (as per Article 3(1)(b) of Regulation (EC) No 141/2000 [3])

In recent years, concomitantly with an increase in the number of applications [4], the conditions proposed for designation are expanding to new areas and becoming increasingly complex, while candidate products may target specific populations or subsets within broader distinct medical entities. This has resulted in an accumulation of experience in the COMP regarding the potential benefits as well as the limitations of biomarker use for regulatory purposes in the field of orphan medicinal products.

This work was produced to present the current perspectives regarding the use of biomarkers in submissions for orphan designation. It is based on an internal EMA-COMP reflection paper produced by an ad-hoc biomarkers working group composed of Members of the COMP and its objectives include:

•Examination of the role of biomarkers in submissions from sponsors seeking Orphan Medicinal Product Designation.

•Presentation and discussion of past submissions to the COMP where biomarkers were used by sponsors in their request for an Orphan Medicinal Product Designation

•Reflection on when and how the COMP has taken biomarkers into consideration when assessing medicinal products.

Table 1 highlights the glossary used in this paper.

**Table 1:** Glossary used in this paper

	
**Biomarker:**	A characteristic that is objectively measured and evaluated as an indicator of normal biologic processes, pathogenic processes, or pharmacologic responses to a therapeutic intervention [5].
**Surrogate endpoints:**	A biomarker intended to substitute for a clinical endpoint. A surrogate endpoint is expected to predict clinical benefit (or harm, or lack of benefit) based on epidemiologic, therapeutic, pathophysiologic or other scientific evidence [5].
**Condition:**	Any deviation(s) from the normal structure or function of the body, as manifested by a characteristic set of signs and symptoms (typically recognized distinct disease or a syndrome) (guideline ENTR/6283/00 Rev3) [2].
**Orphan Condition:**	the condition as defined above that meets the criteria defined in Art.3 of Regulation (EC) No 141/2000 (guideline ENTR/6283/00 Rev3) [2].
**Personalised Medicine:**	A medicine targeted to individual patients, based on their genetic characteristics [6].

### Use of biomarkers in sub-setting and defining a valid sub-set for the purpose of orphan designation

The COMP follows certain guidance documents to assist it in evaluating submissions for Orphan Medicinal Product Designation. One of the principle guidance documents of this nature is the section on General requirements and Special considerations for the description of the condition in an application for orphan designation of Guideline ENTR 6283/00 Rev.03 [2]. Key concepts pertaining to the proposed condition for designation are reproduced from this guideline in the Table 2.

**Table 2:** General requirements and special considerations for the description of the condition in an application for orphan designation reproduced from the guideline ENTR 6283/00 Rev 03

**General requirements**	
(a)	The characteristics defining a distinct condition should determine a group of patients in whom development of a medicinal product is plausible, based on the pathogenesis of the condition and pharmacodynamic evidence and assumptions.
(b)	Recognised distinct medical entities would generally be considered as valid conditions. Such entities would generally be defined in terms of their specific characteristics, e.g. pathophysiological, histopathological, clinical characteristics.
(c)	Different degrees of severity or stages of a disease would generally not be considered as distinct conditions.
The fact that a subset of patients exists in whom the medicinal product is expected to show a favourable benefit/risk (as defined in the proposed therapeutic indication) would generally not be sufficient to define a distinct condition.	
**Special considerations**	
(a)	Considering the above general requirements, convincing arguments would need to be presented to justify the medical plausibility of any proposed subset and the rationale for excluding the larger population. A subset of a disease which, when considered as a whole, has a prevalence greater than 5 in 10,000, could be considered a valid condition if patients in that subset present distinct and unique evaluable characteristic(s) with a plausible link to the condition and if such characteristics are essential for the medicinal product to carry out its action. In particular, the pathophysiological characteristics associated with this subset should be closely linked to the pharmacological action of the medicinal product in such a way that the absence of these characteristics will render the product ineffective in the rest of the population.
(b)	Patients may be affected by more than one condition. Generally the intersection of two (or more) concomitant conditions would not be considered as a valid condition. However, it could be acceptable, if such intersection resulted in a certain new evaluable characteristic essential for the pharmacological effect and the medical outcome.
(c)	Exceptionally, the need for a particular treatment modality (regardless of underlying diseases) can be considered as a valid criterion to define a distinct condition.

Typically only recognised distinct medical entities are considered as valid conditions for designation, while sub-sets are only exceptionally considered. Proposals of subsets based on biomarkers are scrutinized inter alia with regards to the following conditions:

•the subset proposed should fall entirely within a distinct medical condition (therefore not spanning populations affected by more than one distinct medical entity)

•there should be a clear delineation of the subset from the entire of the population (in the context of this paper: a well-defined biomarker)

•the subset should have a “plausible link to the condition” and

•the subset should be “closely linked to the pharmacological action of the medicinal product in such a way that the absence of these characteristics will render the product ineffective in the rest of the larger population with the same condition”

In particular with regards to the requirement of “plausible link to the condition”, this is to be interpreted as a limitation to the category of subsets: the subset may not refer to features that do not pertain directly to the condition or disease, thereby limiting the never-ending potential sub-setting based e.g. on immunological or genomic features of the individual patients and not of the underlying disease.

The requirement of “exclusion of effects outside the proposed subset” is equally important, and involves a vigorous assessment of the pharmacodynamics of the product to ensure that in the excluded population (in the context of this paper the population outside the biomarker-defined subset) the product will not have any favourable pharmacodynamic effect. To illustrate these limitations in the use of biomarker for the purpose of proposing subsets as valid conditions for designation, specific examples are presented below. These were selected on the basis of clarity and usefulness for the points discussed in this paper.

### The limitations of biomarker use in defining acceptable indications for orphan designation

There is a number of unsuccessful cases of orphan applications evaluated by the COMP, which involved biomarkers used to define specific orphan conditions. The justification of “a plausible link to the proposed orphan condition” and the “exclusion of effects” in the wider non-orphan population have proven to be the key drawbacks in these applications. Both of these points are illustrated in the actual case studies elaborated below. As only one of these products was not withdrawn prior to the final opinion from the COMP, the detailed aspects of confidential nature are not discussed in detail.

### Limitations based on the “plausible link to the condition”

The first example pertains to a case discussed in 2013, involving a therapy proposed for treatment of non-small-cell lung cancer in patients expressing HLA-A2.

The Committee considered that in particular the “plausible link to the condition” (Table 2) was not met, even though the “exclusion of effects” could be considered acceptable. It was agreed that HLA-A2 pertained to the immune system of an individual and was not a specific characteristic of a distinct medical condition for which the proposed product is applied for designation. The biomarker in this case was focusing not on the condition but on the individual patient characteristics: the COMP was of the opinion that patients expressing HLA-A2 were actually sub-setted based on their immune system status and the biomarker did not constitute a specific distinct form of non-small-cell lung cancer that could define an orphan condition. The population of non-small-cell lung cancer expressing HLA-A2 was not considered acceptable as an orphan indication.

### Limitations based on the “exclusion of effects”

Two unsuccessful applications are discussed herein, one of which reached the point of formal adoption of a negative opinion that has been made public.

a. In the first case discussed in 2012, a product was proposed for treatment of P-gp positive breast cancer. The sponsor was proposing that P-gp positive breast cancer could be considered a distinct medical condition under the orphan legislation as this breast cancer population is multidrug resistant due to clinically relevant high level of P-gp. The sponsor in the application further clarified that the targeted population covers only 25% of P-gp positive breast cancers, as this is the proportion they expected to be multidrug resistant. The COMP noted that P-gp may be found in close to 100% of breast cancer patients, and that the product could have an effect in a much larger population group than the specific subset proposed by the sponsor as being a distinct orphan medical condition. The COMP considered the 25% cut-off point to be arbitrary and unsupported by current clinical evidence to support the claim that this was a distinct orphan medical condition. The Committee concluded that the condition applied for (treatment of P-gp positive breast cancer) is not a valid subset for orphan designation.

b. Another interesting case was discussed in 2011, pertaining to the application of a product for the treatment of ALK-positive non-small cell lung cancer. In that case the sponsor was proposing that the subset of “ALK-positive non small cell lung cancer” could be considered as a distinct orphan medical condition. This subset is characterised by a gene fusion between the anaplastic lymphoma kinase (ALK) gene and echinoderm microtubule-associated protein-like 4 (EML4), leading to expression of aberrant ALK receptors on the malignant cells. The proposed product for designation was claimed to be a specific inhibitor of ALK. However during the evaluation process it was noted that this product was also an inhibitor of the c-Met/HGFR receptor tyrosine kinase. The mixed nature of product actions established the basis that the proposed sub-setting was not justified since some effects may be anticipated in patients not bearing mutations or ALK overexpression.

### Use of biomarkers in the justification of medical plausibility and significant benefit at orphan designation

As per article 3 of Regulation (EC) No. 141/2000 the sponsor has to establish that the product is “intended for the diagnosis prevention or treatment” of a condition. This intention to diagnose, prevent or treat is examined under the broader term of “medical plausibility” in the proceedings of the COMP, and the relevant guidelines [2].

In addition, significant benefit in Article 3(1)(b) of Regulation (EC) No. 141/2000 requires that in the case where a satisfactory method of diagnosis, prevention or treatment of the condition exists, the sponsor has to establish the medicinal product will be of *significant benefit* to those affected by the condition.

Significant benefit (SB) is further defined in Article 3 of Commission regulation EC 847/2000 as “… a clinically relevant advantage or a major contribution to patient care” [7]. As per commission communication on regulation (EC) No141/2000, “…the justification for significant benefit is likely to be made on assumptions of benefit by the applicant. In all cases the Committee on Orphan Medicinal Products (COMP) is required to assess whether or not these assumptions are supported by available data/evidence supplied by the applicant” [8]. This is also discussed in guideline ENTR/6283/00 Rev 3 section D page 11/13 where it is stated, “…significant benefit should be based on well justified assumptions. Assumptions of potential benefit(s) should be plausible and where possible based on sound pharmacological principles. Preclinical data and preliminary clinical information may be added as supportive evidence. In general a demonstration of potentially greater efficacy, an improved safety profile, and/or more favourable pharma cokinetic properties than existing methods may be considered to support the notion of significant benefit. Other compliance-promoting features or evidence to show fewer interactions with food or other medicinal products, where these are relevant may also be considered” [2].

In order to justify the rationale for the intention to prevent, treat or diagnose, and for the justification of a clinically relevant advantage in case other satisfactory methods exist, biomarkers have been used as surrogate endpoints, without major issues with their use having been identified so far. The sponsor may use them, as long as it justifies that the models or clinical settings are relevant for the proposed condition, and that the endpoints studied are appropriate. A long list of examples, can be found in the monthly release of the COMP plenary meeting minutes that are being made public by the European Medicines Agency since September 2012.

## Discussion

In the context of orphan regulatory procedures, the single major challenge in biomarker use has been the sub-setting of broader medical entities in order to define a valid orphan condition for designation.

With reference to the guideline on the format and content of the applications for designation, patients in a proposed sub-set have to present distinct and unique pathophysiological, histological, aetiological and clinical symptoms which are evaluable characteristic(s) with a plausible link to the proposed orphan condition and the biomarker characteristics should be essential for the medicinal product to carry out its action. Consequently two main requirements in the sub-setting of broader medical entities for the purpose of proposing orphan conditions emerge: 1) establishing a plausible link to the broader sub-setted condition and 2) demonstrating clearly that the presence or absence of the biomarker has a one-to-one relationship with the presence or absence of the drug to execute its pharmacodynamic activities.

The “plausible link” requirement may be viewed as an argument against proposing subsets of non-rare conditions in the era of personalized medicine: when the biomarker refers not to the condition but to the individual patient this link is not preserved. At the same time, it may be viewed as a guardian of evolution of our scientific understanding: when the biomarkers refer to the condition then the link is confirmed, and this is of particular importance in cases when biomarkers redefine the classification of medical entities as our understanding evolves (diagnostic biomarkers). The specific nature of biomarkers may be associated with particular regulatory difficulties. For example, biomarkers used in the context of receptors on cancer cells, present a particular challenge as their detection capability may evolve by further development of newer detection assays. The tests are in time rendered more specific and sensitive and could help in identifying populations who will respond more effectively to the therapy but who do not necessarily represent a distinct patient population under the definition of the orphan legislation. Another example, indicative of the complexity is the tyrosine kinase area: the fact that an applicant for an orphan designation does not identify cross reactivity with other targets does not prevent that a wider investigation and testing of more cell lines or biopsies may eventually detect some cross reactivity. This would again challenge the volume of the subset compared to the underlying broader distinct medical entity. These examples illustrate the problem of unequivocally setting thresholds for definitions of acceptable sub-sets based on biomarkers.

To take the argument a bit further, it is also becoming apparent that our scientific understanding underpinning the usefulness of biomarkers has been changing and evolving over time. Indicatively in neurofibromatosis with children it was presumed that tamoxifen should be used if the tumours were oestrogen receptor positive. But later it appeared that the product was not working only via the receptor and that also oestrogen receptor negative patients could benefit. A similar case seems to be with the KRAS mutation in colorectal cancer. Originally it was thought cetuximab would only be working in wild type KRAS patients but there are some other studies indicating that at least some KRAS mutation patients will benefit as well.

Lastly, an interesting point is the evolution of the standards in the proceedings of the COMP, in the context of the increasing complexity of medicinal products reviewed and the recent advancements in scientific and medical research. Compared to the early days of the Committee, this has crystallised into shifting the focus from mainly the “exclusion of effects outside the subset”, to equally examining both the “exclusion of effects” and the “plausible link” principles with equal impact. This is clearly reflected in the examples described above.

## Conclusions

•Sub-setting of medical entities to define a valid orphan condition and justification of the criteria for orphan designation have been the main areas in which the COMP has used biomarkers and clinical surrogate endpoints.

•If biomarkers are to be taken into account for the proposal of a sub-set of a broader distinct medical entity as a valid condition for designation, a decisive factor is the “plausible link to the condition”. The biomarker should not refer, in that case, to features external to the broader distinct medical entity.

•In addition, if biomarkers are to be taken into account for sub-setting, the anticipated presence or absence of any pharmacodynamic effect, in either preclinical or clinical settings, is to be justified and supplemented by a convincing scientific argumentation and data justifying that the product will not work in the excluded patients.

•Even though biomarkers can define a valid sub-set of a condition acceptable for designation, there is still a need to demonstrate medical plausibility and significant benefit in the defined condition.

•As per the orphan regulation, at the time of marketing authorisation the criteria for orphan designation are reassessed, which also means that the validity of the used biomarker(s) has to be proven at that time.

## Abbreviations

EMA: European Medicines Agency; COMP: Committee of Orphan Medicinal Products; HLA: Human leukocyte antigen; P-gp: P-glycoprotein; ALK: Anaplastic lymphoma kinase; KRAS: v-Ki-ras2 Kirsten rat sarcoma viral oncogene homolog.

## Competing interests

No competing interests are declared.

## Authors’ contributions

All authors contributed equally to this work. All authors read and approved the final manuscript.
